# Associations of Two Obesity-Related Single-Nucleotide Polymorphisms with Adiponectin in Chinese Children

**DOI:** 10.1155/2017/6437542

**Published:** 2017-03-15

**Authors:** Lijun Wu, Liwang Gao, Xiaoyuan Zhao, Meixian Zhang, Jianxin Wu, Jie Mi

**Affiliations:** ^1^Department of Epidemiology, Capital Institute of Pediatrics, Beijing, China; ^2^Department of Biochemistry, Capital Institute of Pediatrics, Beijing, China

## Abstract

*Purpose*. Genome-wide association studies have found two obesity-related single-nucleotide polymorphisms (SNPs), rs17782313 near the melanocortin-4 receptor (*MC4R*) gene and rs6265 near the brain-derived neurotrophic factor (*BDNF*) gene, but the associations of both SNPs with other obesity-related traits are not fully described, especially in children. The aim of the present study is to investigate the associations between the SNPs and adiponectin that has a regulatory role in glucose and lipid metabolism. *Methods*. We examined the associations of the SNPs with adiponectin in Beijing Child and Adolescent Metabolic Syndrome (BCAMS) study. A total of 3503 children participated in the study. *Results*. The SNP rs6265 was significantly associated with adiponectin under an additive model (*P* = 0.02 and 0.024, resp.) after adjustment for age, gender, and BMI or obesity statuses. The SNP rs17782313 was significantly associated with low adiponectin under a recessive model. No statistical significance was found between the two SNPs and low adiponectin after correction for multiple testing. *Conclusion*. We demonstrate for the first time that the SNP rs17782313 near *MC4R* and the SNP rs6265 near *BDNF* are associated with adiponectin in Chinese children. These novel findings provide important evidence that adiponectin possibly mediates MC4R and BDNF involved in obesity.

## 1. Introduction

In recent years, adult and childhood obesity has reached epidemic proportions globally [1], and the increasing prevalence of obesity is a major threat to public health. Childhood obesity strongly predisposes to adult obesity and diseases including type 2 diabetes, cardiovascular disease, and hypertension [2–5].

Previously, multiple single-nucleotide polymorphisms (SNPs) related to obesity have been identified by genome-wide association studies [6–8], and large population-based studies have been conducted to confirm common genetic variants that contribute to obesity [9–11]. However, the associations of those SNPs with other obesity-related traits are not fully described, especially in children.

Adiponectin is an adipocyte-derived hormone in inverse proportion to the body mass index (BMI) and appears to have a regulatory role in glucose and lipid metabolism [12, 13]. Recent studies indicate that adiponectin has anti-inflammatory, antiatherogenic, and insulin-sensitizing effects [14].

Evidence from our previous study suggested that two SNPs (rs17782313 near the melanocortin-4 receptor (*MC4R*) gene and rs6265 near the brain-derived neurotrophic factor (*BDNF*) gene) were significantly associated with BMI and obesity [10], but the molecular mechanisms are not understood. *MC4R* and *BDNF* were found to be highly expressed in the hypothalamus, which suggests that they may play important roles in the central nervous system in eating behavior [15, 16]. Golden et al. reported that the BDNF level was inversely correlated with adiponectin in adult males [17]. There has been no evidence that the SNPs in or near *MC4R* or *BDNF* are associated with adiponectin. We investigated the associations between the SNP rs17782313 near *MC4R* and the SNP rs6265 near *BDNF* with adiponectin in the cohort. We genotyped the two SNPs in Chinese children who participated in the population-based Beijing Child and Adolescent Metabolic Syndrome (BCAMS) study [18]. The present study attempts to provide an analysis of epidemiological and genetic data towards the possible mechanism of the role of *MC4R* or *BDNF* in obesity.

## 2. Methods

### 2.1. Study Population

Subjects were recruited from the BCAMS study, a cross-sectional population-based survey carried out in 2004 [18]. The survey included a questionnaire, medical examination, anthropometric measurement, and finger capillary blood tests in a representative sample (*n* = 19593, 50% boys) of children in Beijing aged 6–18 years in 2004. Anthropometric measurements included weight, height, waist circumference, and fat-mass percentage. Within this large group of children, 1229 obese, 655 overweight, and 1619 normal-weight children were randomly recruited and diagnosed by using the Chinese age- and sex-specific BMI cutoffs [19]. Venipuncture blood samples were collected for genotyping. The BCAMS study was approved by the ethics committee of Capital Institute of Pediatrics. We obtained written informed consent from parents or guardians.

### 2.2. Measurement of Biochemical Analyses and Genotyping

The levels of adipocytokines were measured by ELISA techniques [20]. Genomic DNA was isolated from peripheral white blood cells using the salt fractionation method. Genotyping of rs17782313 and rs6265 was conducted using the TaqMan Allelic Discrimination Assay with the GeneAmp 7900 Sequence Detection System (Applied Biosystems, Foster City, CA, USA) and with the TaqMan probes (C__32667060_10 and C__11592758_10). The genotyping call rates for both SNPs were greater than 98%. We repeated 70 samples randomly for each SNP to validate the accuracy of genotyping and observed 100% concordance between the results of the two tests. We also sent 30 samples to direct sequencing and observed 100% concordance between two genotyping methods.

### 2.3. Statistical Analyses

Continuous variables were presented as mean ± standard deviation (SD), and categorical variables were presented as percentages. Hardy-Weinberg equilibrium was assessed using the chi-square test. Adjusted odds ratios (ORs) for low adiponectin were performed by logistic regression with genotypes, age, gender, and BMI or obesity statuses as the independent variables. A logistic regression model was used to investigate the interaction between rs17782313 and rs6265 on low adiponectin. The data were analysed using SPSS statistical software. *P* < 0.05 was used to indicate statistically significant differences. False discovery rate (FDR) approach was used to correct for multiple testing. In brief, the stringent *P* value was considered statistically significant only if it was less than 0.05. Power calculation was performed using Quanto software (http://hydra.usc.edu/gxe/).

## 3. Results

The basic characteristics of the study participants are summarized in supplementary Table 1 available online at https://doi.org/10.1155/2017/6437542. We genotyped the SNP rs17782313 near *MC4R* and the SNP rs6265 near *BDNF* in the cohort, and the genotypes of the SNPs were tested to be in Hardy-Weinberg equilibrium (*P* = 0.978 and 0.334, resp.). Associations of rs17782313 and rs6265 with adiponectin levels are shown in Table 1. There were statistically significant associations of rs17782313 and rs6265 with adiponectin levels after adjustment for age and gender. As both SNPs were significantly associated with BMI, we also adjusted BMI or obesity statuses besides age and gender. After correction for multiple testing, the SNP rs6265 was significantly associated with adiponectin under an additive model (*P* = 0.02 and 0.024, resp.) after adjustment for age, gender, and BMI or obesity statuses.

Table 2 shows the associations of the two SNPs with low adiponectin diagnosed by less than the 25 percentile of the participant with the same age and gender. The SNP rs17782313 was significantly associated with low adiponectin under a recessive model after adjustment for age, gender, and BMI or obesity statuses (OR = 0.700, 95% confidence interval (CI): 0.490–1.000, *P* = 0.050; OR = 0.699, 95% CI: 0.491–0.996, *P* = 0.048, resp.). However, no statistical significance was found between the two SNPs and low adiponectin after correction for multiple testing (Table 2). Studies with greater sample size are needed to confirm these associations.

We also investigated the interaction between rs17782313 and rs6265 on low adiponectin (supplementary Table 2). There was no statistically significant interaction between rs17782313 and rs6265 on low adiponectin.

## 4. Discussion

In this study, we examined the SNP rs17782313 near *MC4R* and the SNP rs6265 near *BDNF* in Chinese children. Our results indicated that the SNP rs6265 was significantly associated with adiponectin after adjustment for age, gender, and BMI or obesity statuses (Table 1), and no statistical significance was found between the two SNPs and low adiponectin after correction for multiple testing (Table 2).

Adiponectin and BMI in the groups with different genotypes of rs17782313 and rs6265 are shown in supplementary Table 3. The mean of adiponectin in the group with CC genotype of rs17782313 is the lowest in the groups with three genotypes of the SNP. Meanwhile, the mean of BMI in the group with CC genotype of rs17782313 is the highest in the groups with three genotypes of the SNP. Similarly, the group with GG genotype of rs6265 has the lowest mean of adiponectin and the highest mean of BMI in the groups with three genotypes of the SNP. This trend is coincident with the known relationship between BMI and adiponectin, and it suggests that C allele of rs17782313 and G allele of rs6265 increase the risk of low adiponectin.

A previous study has shown that the plasma BDNF level was inversely correlated with adiponectin in adult males [17]. We did not investigate the plasma BDNF level in the cohort, and this study may not provide direct evidence that the SNP rs6265 influences the BDNF level.

As the two SNPs are involved in the leptin/melanocortin pathway, we also investigated the associations of the two SNPs with leptin levels. There was no statistical significance between the two SNPs and leptin levels after adjustment for age, gender, and BMI or obesity statuses (data not shown). Studies with greater sample size are needed to confirm the associations.

Moreover, Dastani et al. identified novel loci for adiponectin levels and their influence on type 2 diabetes and metabolic traits and nicely complemented our study [21].

Our study demonstrate for the first time that the SNP rs17782313 near *MC4R* and the SNP rs6265 near *BDNF* are associated with adiponectin in Chinese children, but this study may not provide direct evidence that the expression of *MC4R* and *BDNF* influences obesity because of the lack of gene expression data.

## 5. Conclusions

We demonstrate for the first time that the SNP rs17782313 near *MC4R* and the SNP rs6265 near *BDNF* are associated with adiponectin in Chinese children. These novel findings provide important evidence that adiponectin may be possibly mediate the process of MC4R and BDNF involved in obesity.

## Conflicts of Interest

The authors declare that there is no conflict of interests regarding the publication of this paper.

## Supplementary Material

The supplemental material contains basic characteristics of study participants (Supplementary Table 1), interaction between rs17782313 and rs6265 on low adiponectin (Supplementary Table 2) and adiponectin and BMI in groups with different genotypes of rs17782313 and rs6265 (Supplementary Table 3).

## Figures and Tables

**Table 1:** Associations of rs17782313 and rs6265 with adiponectin.

SNP	Gene	Additive model	*N*	Adiponectin (mg L^−1^)	Stringent *P* value^a^	Stringent *P* value^b^	Stringent *P* value^c^
rs17782313	*MC4R*	CC	186	11.63 ± 7.65			
		CT	1230	12.59 ± 7.25			
		TT	2028	13.02 ± 7.48	**0.007**	0.263	0.222
rs6265	*BDNF*	GG	971	12.31 ± 6.91			
		GA	1744	12.72 ± 7.45			
		AA	733	13.63 ± 7.92	**0.002**	**0.02**	**0.024**

MC4R: the melanocortin-4 receptor gene; BDNF: the brain-derived neurotrophic factor gene.

^a^Adjusted for age and gender.

^b^Adjusted for age, gender, and BMI.

^c^Adjusted for age, gender, and obesity statuses.

The values highlighted in bold indicate that the associations showed statistical significance.

Adiponectin is expressed as the mean ± standard deviation.

**Table 2:** Associations of rs17782313 and rs6265 with low adiponectin under the additive model.

SNP	Gene	Adjusted for age and gender				Adjusted for age, gender, and BMI				Adjusted for age, gender, and obesity statuses			
		OR	95% CI	Stringent *P* value	Power	OR	95% CI	Stringent *P* value	Power	OR	95% CI	Stringent *P* value	Power
rs6265	*BDNF*	0.904	0.799–1.023	0.111	0.956	0.933	0.822–1.060	0.574	0.713	0.938	0.826–1.064	0.319	0.644
rs17782313	*MC4R*	0.848	0.736–0.977	**0.044**	0.996	1.04	0.890–1.220	0.604	0.210	0.909	0.786–1.050	0.380	0.778

OR: odds ratio; CI: confidence interval.

The value highlighted in bold indicates that the associations showed statistical significance.
