# Supplementary material for: Associations of Two Obesity-Related Single-Nucleotide Polymorphisms with Adiponectin in Chinese Children
Source: Int J Endocrinol. 2017 Mar 15;2017:6437542. doi: 10.1155/2017/6437542 (PMC5370521; doi:10.1155/2017/6437542)
Supplement: Supplementary file 1 — The supplemental material contains basic characteristics of study participants (Supplementary Table 1), interaction between rs17782313 and rs6265 on low adiponectin (Supplementary Table 2) and adiponectin and BMI in groups with different genotypes of rs17782313 and rs6265 (Supplementary Table 3). [file 6437542.f1.doc]

**Supplementary tables**

Supplementary TABLE 1: Basic characteristics of study participants.

| Characteristics | All |
| --- | --- |
| N | 3503 |
| Male (%) | 50.8 |
| Age (years) | 12.4±3.1 |
| BMI (kg m^-2^) | 21.9±4.9 |
| Weight statues (%) |  |
| Obese | 35.0 |
| Overweight | 18.5 |
| Normal weight | 46.0 |
| Leptin (µg L^-1^) | 10.03±11.41 |
| Adiponectin (mg L^-1^) | 12.79±7.42 |
| Resistin (µg L^-1^) | 18.41±24.13 |

BMI, body mass index.

Data are presented as mean ±standard deviation, or percentages of subjects, as appropriate. The subjects were diagnosed by using the Chinese age- and sex-specific BMI cutoffs.^1^

^1^ Ji CY, Working Group on Obesity in China. Report on childhood obesity in China (1)--body mass index reference for screening overweight and obesity in Chinese school-age children. *Biomed Environ Sci* 2005;18:390-400.

Supplementary TABLE 2: Interaction between rs17782313 and rs6265 on low adiponectin.

| SNP | Adjusted for age and gender | | |  | Adjusted for age ,gender and BMI | | |  | Adjusted for age ,gender and obesity statues | | |
| --- | --- | --- | --- | --- | --- | --- | --- | --- | --- | --- | --- |
|  | *β* | 95% CI | *P*-*value* |  | *β* | 95% CI | *P-value* |  | *β* | 95% CI | *P-value* |
| rs17782313*rs6265 | 0.950 | 0.775-1.164 | 0.620 |  | 0.958 | 0.778-1.18 | 0.688 |  | 0.96 | 0.780-1.179 | 0.691 |

CI, confidence interval.

Supplementary TABLE 3: Adiponectin and BMI in groups with different genotypes of rs17782313 and rs6265.

| SNP | Gene | Genotype | N | Adiponectin (mg L^-1^) | BMI (kg m^-2^) |
| --- | --- | --- | --- | --- | --- |
| rs17782313 | *MC4R* | CC | 186 | 11.63±7.65 | 23.11±5.20 |
|  |  | CT | 1230 | 12.59±7.25 | 22.17±4.89 |
|  |  | TT | 2028 | 13.02±7.48 | 21.63±4.92 |
|  |  |  |  |  |  |
| rs6265 | *BDNF* | GG | 971 | 12.31±6.91 | 22.25±4.99 |
|  |  | GA | 1744 | 12.72±7.45 | 21.88±4.90 |
|  |  | AA | 733 | 13.63±7.92 | 21.54±4.93 |

*MC4R*, the melanocortin-4 receptor gene; *BDNF*, the brain-derived neurotrophic factor gene.

Adiponectin are expressed as the mean±standard deviation.
