# Supplementary material for: Associations of Two Obesity-Related Single-Nucleotide Polymorphisms with Adiponectin in Chinese Children
Source: Int J Endocrinol. 2017 Mar 15;2017:6437542. doi: 10.1155/2017/6437542 (PMC5370521; doi:10.1155/2017/6437542)
Supplement: Supplementary file 2 [file 6437542.f2.pdf]

Supplementary tables

Supplementary TABLE 1: Basic characteristics of study participants.

Characteristics	All
N	3503
Male (%)	50.8
Age (years)	12.4±3.1
BMI (kg m^{-2})	21.9±4.9
Weight statues (%)	
Obese	35.0
Overweight	18.5
Normal weight	46.0
Leptin (μg L^{-1})	10.03±11.41
Adiponectin (mg L^{-1})	12.79±7.42
Resistin (μg L^{-1})	18.41±24.13

BMI, body mass index.

Data are presented as mean ±standard deviation, or percentages of subjects, as appropriate. The subjects were diagnosed by using the Chinese age- and sex-specific BMI cutoffs.[1]

[1] Ji CY, Working Group on Obesity in China. Report on childhood obesity in China (1)--body mass index reference for screening overweight and obesity in Chinese school-age children. *Biomed Environ Sci* 2005;18:390-400.

Supplementary TABLE 2: Interaction between rs17782313 and rs6265 on low adiponectin.

SNP	Adjusted for age and gender			Adjusted for age ,gender and BMI			Adjusted for age ,gender and obesity statues		
	β	95% CI	*P-value*	*β*	95% CI	*P-value*	*β*	95% CI	*P-value*
rs17782313*rs6265	0.950	0.775-1.164	0.620	0.958	0.778-1.18	0.688	0.96	0.780-1.179	0.691

CI, confidence interval.

Supplementary TABLE 3: Adiponectin and BMI in groups with different genotypes of rs17782313 and rs6265.

SNP	Gene	Genotype	N	Adiponectin (mg L^{-1})	BMI (kg m^{-2})
rs17782313	*MC4R*	CC	186	11.63±7.65	23.11±5.20
		CT	1230	12.59±7.25	22.17±4.89
		TT	2028	13.02±7.48	21.63±4.92
rs6265	*BDNF*	GG	971	12.31±6.91	22.25±4.99
		GA	1744	12.72±7.45	21.88±4.90
		AA	733	13.63±7.92	21.54±4.93

MC4R, the melanocortin-4 receptor gene; *BDNF*, the brain-derived neurotrophic factor gene.

Adiponectin are expressed as the mean±standard deviation.
